# Synthesis and Characterization of Ferroelectric Liquid Crystalline Organosiloxanes Containing 4-(4-undecanyloxy bi-phenyl-1-carboxyloxy)phenyl (2*S*,3*S*)-2-chloro-3-methylvalerate and 4-(4-undecanyloxybenzoyloxy)biphenyl (2*S*,3*S*)-2-chloro-3-methylvalerate

**DOI:** 10.3390/ijms141121306

**Published:** 2013-10-25

**Authors:** Chih-Hung Lin

**Affiliations:** 1 Center for General Education, Chang Gung University of Science and Technology, No.261, Wenhua 1st Rd., Guishan Township, Taoyuan County 33303, Taiwan; 2 Research Center for Industry of Human Ecology, Chang Gung University of Science and Technology, No.261, Wenhua 1st Rd., Guishan Township, Taoyuan County 33303, Taiwan

**Keywords:** siloxane, spontaneous polarization, ferroelectric liquid crystal

## Abstract

A series of new organosiloxane ferroelectric liquid crystalline (FLC) materials have been synthesized, and their mesomorphic and physical properties have been characterized. Four new disiloxanes and trisiloxanes, containing biphenyl 4-hydroxybenzoate and phenyl 4-hydroxybiphenylcarboxylate as mesogenic units and eleven methylene unit as spacers and (2*S*,3*S*)-2-chloro-3-methylvalerate unit as chiral end groups. The molecule, using three phenyl ring as a mesogenic unit, formulates much wider liquid crystalline phase temperature ranges than that of a two phenyl ring unit. The phenyl arrangement differences of mesogenic unit result in the greater differences of the liquid crystal phase formation. The siloxane molecule induction is helpful to the more regular smectic phase formation and smectic phase stabilization, such as chiral S_C_ (S_C_*) and S_B_ phases. The siloxane molecule is helpful to reduce the phase transition temperature and broaden the liquid crystal temperature range of the S_C_* phase and, simultaneously, it will not induce chain crystallization phenomenon and dilute the *P*s value. The synthesis and characterization of the new FLCs materials, which exhibit a room temperature S_C_* phase and higher spontaneous polarization are presented.

## 1. Introduction

Organosiloxane low molar mass (LMM) liquid crystals have attracted considerable interest because their electro-optic properties are similar to those of classical LMM liquid crystals, demonstrating fast switching times in the nematic [1], S_A_[2], and S_C_* phases [3–5]. Ferroelectric liquid crystalline (FLC) materials, exhibiting a room temperature S_C_* phase and low melting temperatures are of great interest to researchers in the electro-optic field [6–11]. They are the basis of a variety of potential devices, ranging from large-area flat-panel displays**,** ultrafast electro-optic modulators, and spatial light modulators [12–16].

FLC molecules consist of a rigid core and end-tail groups with a chiral center located on one or both groups. The mesogenic moiety usually uses a benzene ring as rigid core, and the number of the benzene ring will influence the temperature range of the liquid crystal. For most FLC molecules, the end-tail groups are hydrocarbon chains. When increasing the hydrocarbon chain lengths of the molecules, the rotational viscosity, as well as the tilt angle of the S_C_* molecules, increase. Increasing the length of the non-polar moiety helps to dilute the transverse dipole moment in the material and thus decrease the value of *Ps*, whereas, the increase of the tilt angle should have a tendency to increase *Ps* and the FLC molecule is also easier to crystallize.

Recently, we have synthesized a series of organosiloxane FLC materials with a biphenyl mesogenic moiety [17]. In this study, we designed and prepared two new series of molecules, which contains two chiral centers and dimethylsiloxyl (or trimethylsiloxyl) substituent groups linked through flexible hydrocarbon chains. The difference between these two series is the sequence of the phenyl rings’ arrangements in the rigid core structure. The substitution of methylsiloxyl groups was expected to enhance the thermal stability and *Ps* value of the FLC materials [18–21].

## 2. Results and Discussion

This study intends to explore two influences: one is the influence of the liquid crystal compound with siloxane end-tail, and the other is the influence from different rigid core arrangements in the mesogenic unit that change the thermal properties of liquid crystalline and liquid crystal mesophase.

**Scheme 1:**
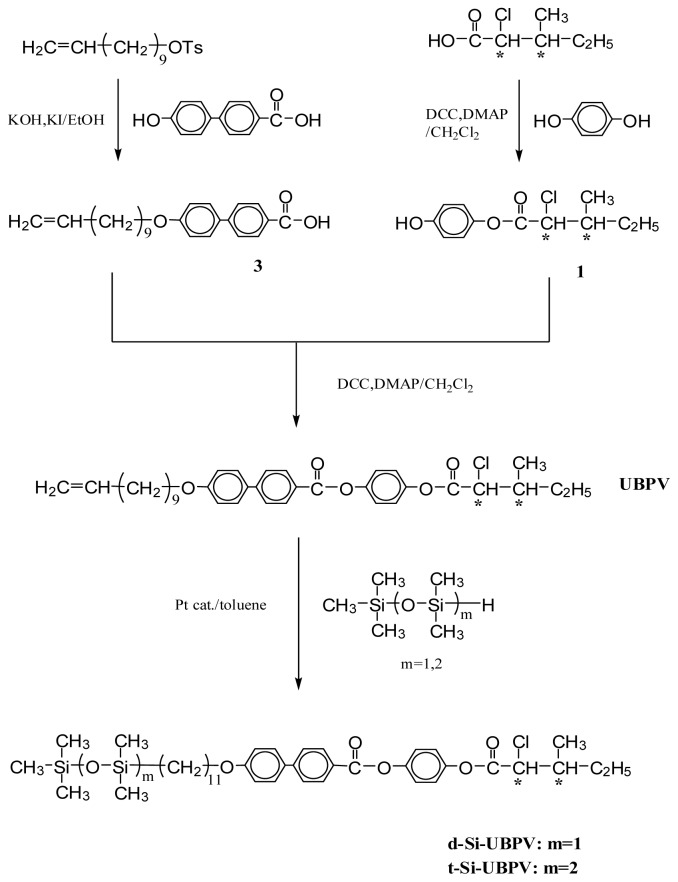
Synthesis of compounds UBPV, d-Si-UBPV, and t-Si-UBPV. (series UBPV) * chiral center.

**Scheme 2:**
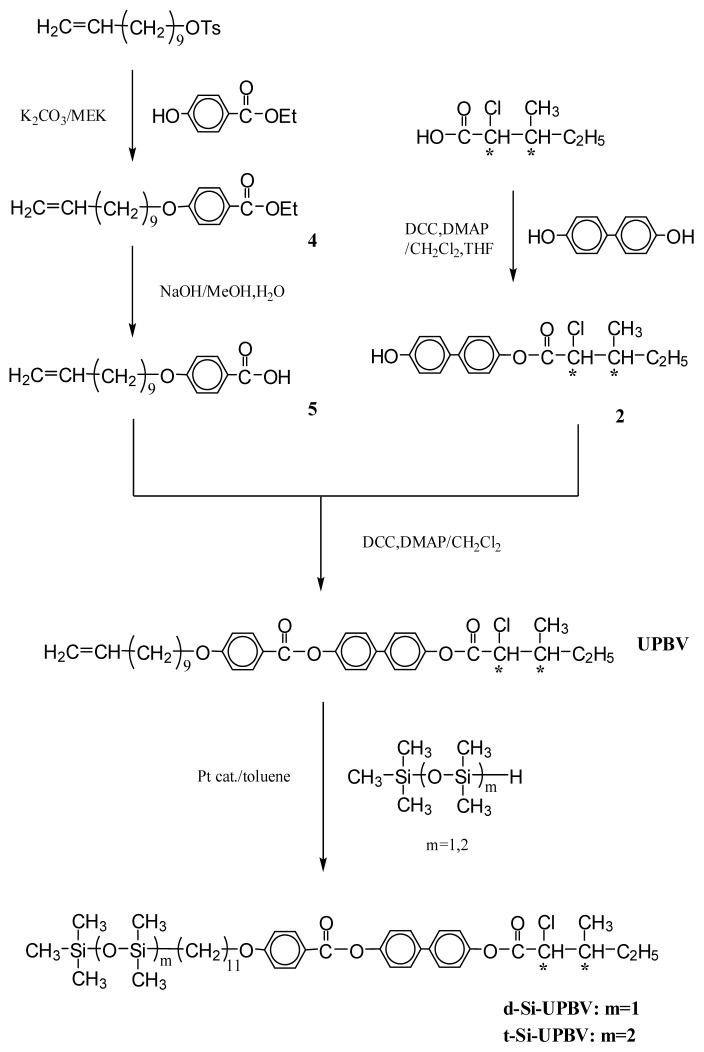
Synthesis of compounds UPBV, d-Si-UPBV, and t-Si-UPBV. * chiral center.

In order to clarify the influences, two series of liquid crystal compounds with different mesogenic moieties have been designed and synthesized (series UBPV and series UPBV). Schemes 1,2 illustrate the synthesis procedure of the reaction intermediates and the liquid crystal compounds, and all the products of these two series were examined by the nuclear magnetic resonance spectrometer to verify the correction of the molecular structure.

In the UBPV series of compounds (UBPV, d-Si-UBPV, and t-Si-UBPV**),** a biphenyl group accompanying a phenyl group are used as the mesogenic units; but in the UPBV series of compounds (UPBV, d-Si-UPBV, and t-Si-UPBV) different phenyl rings arrangement in order are applied to explore the influence of different rigid core in the liquid crystal compounds. In addition, three different end-tails, which are ethylene, dimethylsiloxyl, and trimethylsiloxyl groups, were incorporated to the core through an eleven-carbon aliphatic spacer in each series.

**Figure 1:**
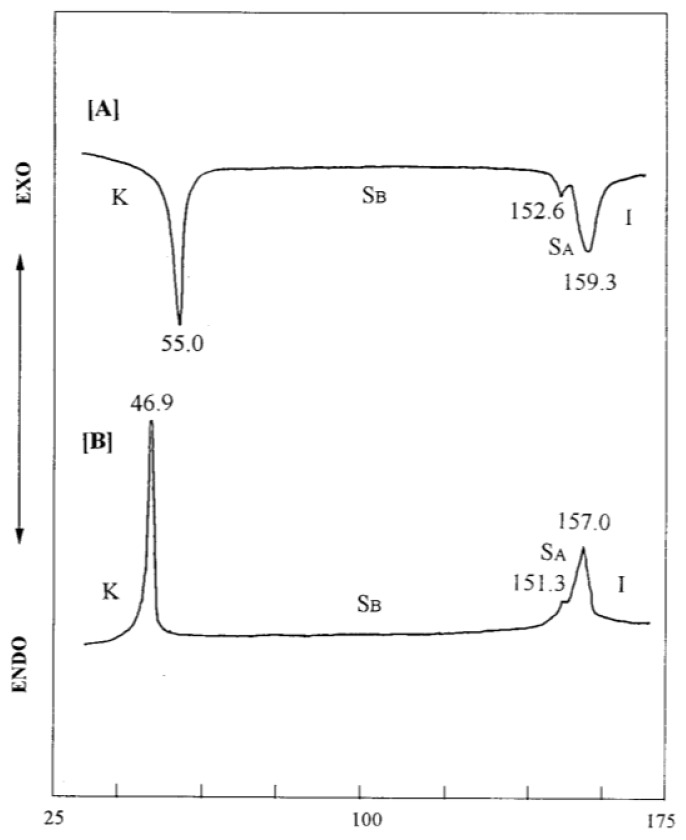
DSC thermograms of compound d-Si-UBPV (10 °C/min) for (**A**) second heating scanning and (**B**) cooling scanning. K = crystal; S_A_ = smectic A; S_B_ = smectic B.

**Figure 2:**
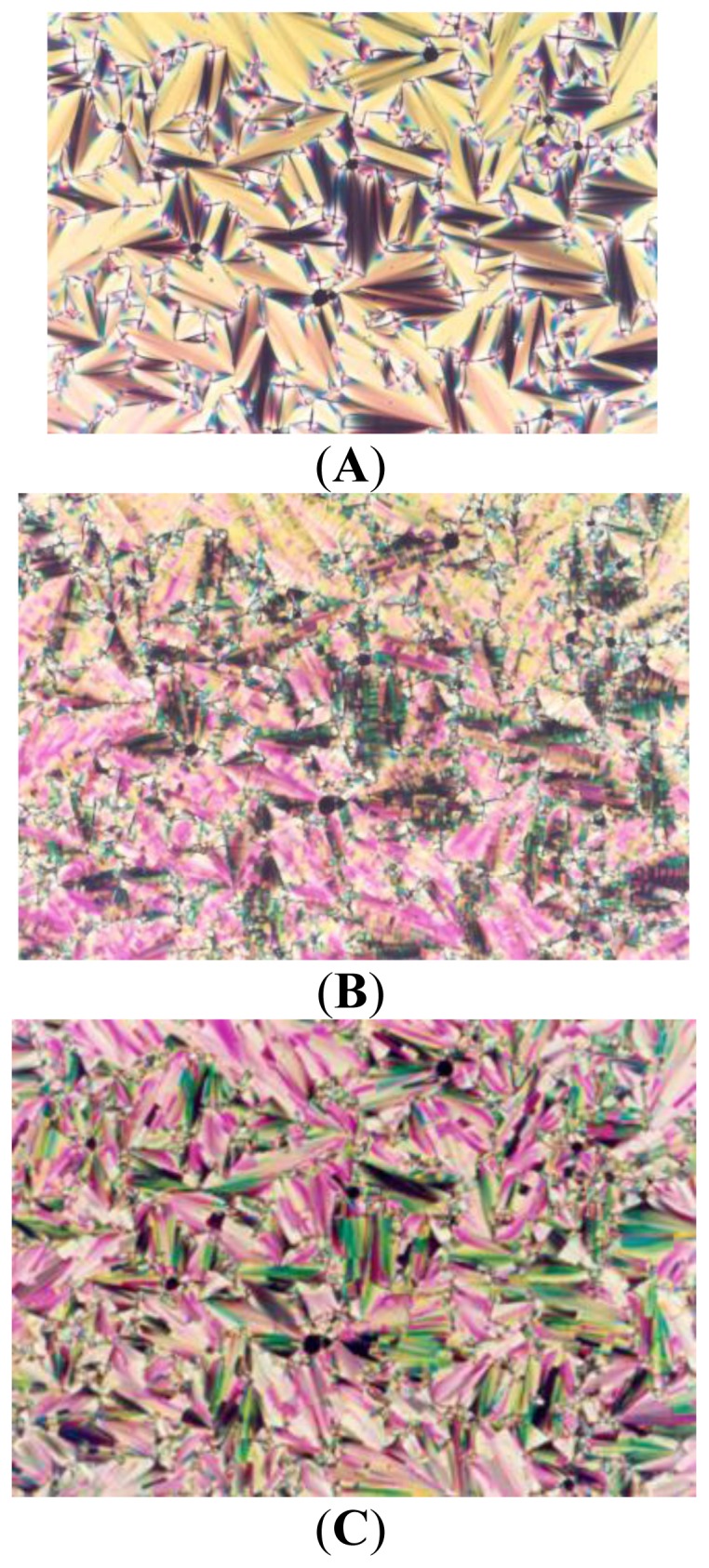
Polarizing optical micrographs (magnification × 320) of compound d-Si-UBPV in (**A**) S_A_ texture obtained after cooling from isotropic phase to 155 °C and (**B**) S_A_ to S_B_ texture obtained after cooling to 151 °C; (**C**) S_B_ texture obtained after cooling to 140 °C.

The thermal and mesomorphic properties of compounds series UBPV and series UPBV were measured using differential scanning calorimeter(DSC) and polarized optical microscope(POM). The phase transition temperatures and enthalpy change of series UBPV compounds are listed in Table 1. As the mesogenic units in the series UBPV compounds, containing three phenyl rings, the biphenyl segment is linked directly to the spacer, but is farther from the chiral group. Surprisingly, this series compounds do not possess a S_C_* phase. In the cooling process, compound UBPV owns an enantiotropic S_A_ phase in which the temperature range is about 37 °C and a monotropic S_B_ phase in which the temperature range is about 10 °C. When siloxane was inducted into UBPV, d-Si-UBPV, and t-Si-UBPV were obtained; they showed an enantiotropic S_B_ phase with a quite larger temperature range of about 100 °C. In addition, the S_A_ phase is diminished and almost overlaps with other liquid crystal mesophases. The DSC analysis diagram of compound d**-**Si-UBPV is depicted in Figure 1. Compound d-Si-UBPV, equipped with a liquid crystalline texture diagram of S_A_ and S_B_ by using POM is shown in Figure 2A,C. Figure 2B depicts the liquid crystalline texture diagram of phase transition process from the S_A_ phase to the S_B_ phase.

Among the UBPV series compounds, t-Si-UBPV has the widest liquid crystalline temperature range (113.7 °C), and the liquid crystalline temperature range of compound d-Si-UBPV is slightly lower. The compound UBPV shows the smallest liquid crystalline temperature range of 46.2 °C. Consequently, the results can verify a fact that siloxane group is able to stabilize the liquid crystalline formation and the more regular smectic formation, such as the S_A_ phase changing to the S_B_ phase.

**Figure 3:**
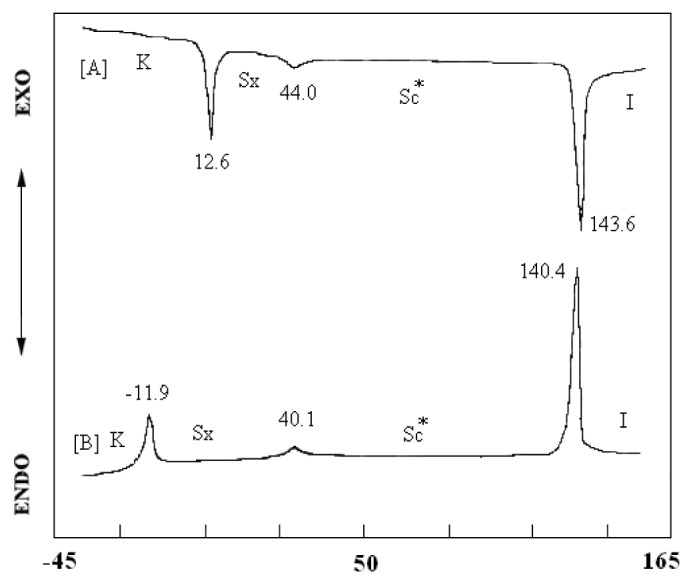
DSC thermograms of compound d-Si-UPBV (10 °C/min) for (**A**) second heating scanning and (**B**) cooling scanning. K = crystal; S_C_* = chiral smectic C; *S_X_* = smectic phase.

The phase transition temperatures and enthalpy change of compounds UPBVs are listed in Table 2. The phase transition of compound UPBV displays both enantiotropic chiral nematic and S_A_ phase. When siloxane was inducted into compound UPBV, the results of compound d-Si-UPBV and t-Si-UPBV were obtained, possessing a liquid crystalline phase with the temperature range of about 152 °C. Furthermore, two situations appear: one is an enantiotropic S_C_* liquid crystal phase with the temperature range of about 100 °C. The other is a more regulated smectic phase, either an S_F_ liquid crystal phase or an S_I_ liquid crystal phase, with a cooling function to lower the transition temperatures of the above (S_F_ or S_I_) liquid crystal phases. Thus, compound UPBV shows a phase transition temperature at 43.4 °C and compounds d-Si-UPBV and t-Si-UPBV show phase transition temperatures at −11.9 °C and −15.4 °C, respectively. Figure 3 depicts the DSC analysis diagram of compound d-Si-UPBV. As a result, it can be summarized that the flexible siloxane molecule induction is helpful in lowering the liquid crystalline phase transition temperature and stabilizing the smectic phase formation, such as chiral nematic phase changing to the S_C_* phase.

The formation of a S_C_* phase was further verified by spontaneous polarization (*Ps*) measurements. Compounds d-Si-UPBV and t-Si-UPBV were filled into a four-milligram thick cell, made of ITO glass, of which the surface was coated in polyimide, which was rubbed in a parallel direction. Voltage (about 5 V/μm) of 50 Hz frequency and 20 Vp (peak voltage) was used, and the measurement area was 0.26 cm^2^. The sample was heated to an isotropic temperature, adding voltage to sample in the cooling process. Measurements of *Ps* value and rotational viscosity were taken every 0.5–1 °C during the S_C_* mesophase range.

**Figure 4:**
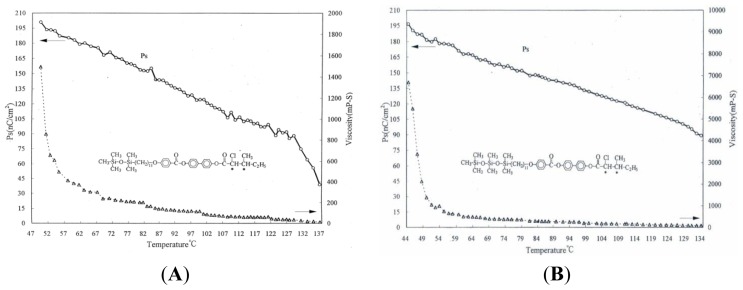
Spontaneous polarization (*Ps*) and viscosity *vs.* temperature for compound (**A**) d-Si-UPBV and (**B**) t-Si-UPBV.

The *Ps* value and rotational viscosity of various compounds have the tendency to drop when the temperatures increases. Figure 4 represents the spontaneous polarization (*Ps*) as a function of temperature for compound d-Si-UPBV. The maximum *Ps* value of compound d-Si-UPBV was about 200 nC/cm^2^ at 50 °C, compound t-Si-UPBV was about 197 nC/cm^2^ at 44 °C.

**Figure 5:**
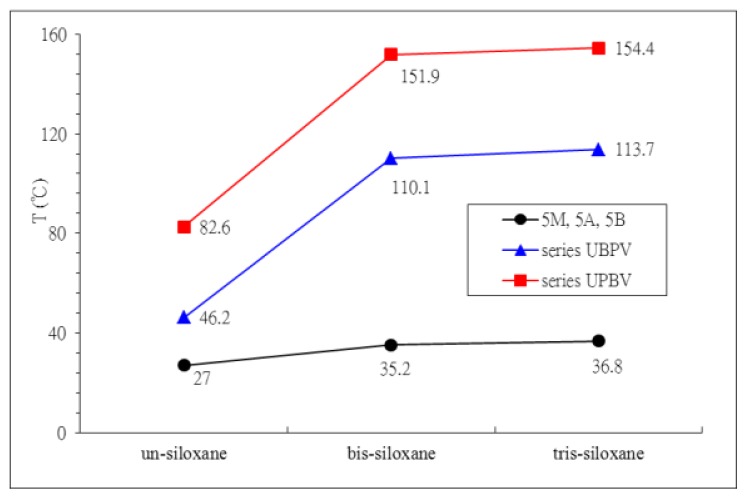
Liquid crystal temperature ranges of 5M, 5A, 5B ^a^, UBPVs, and UPBVs. ^a^ The chemical structure of 5M, 5A, and 5B [17].

**Figure 6:**
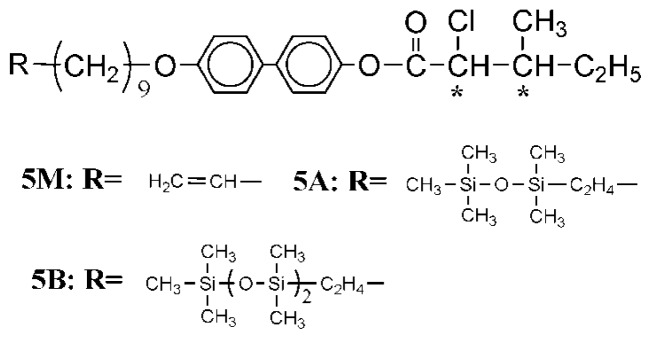

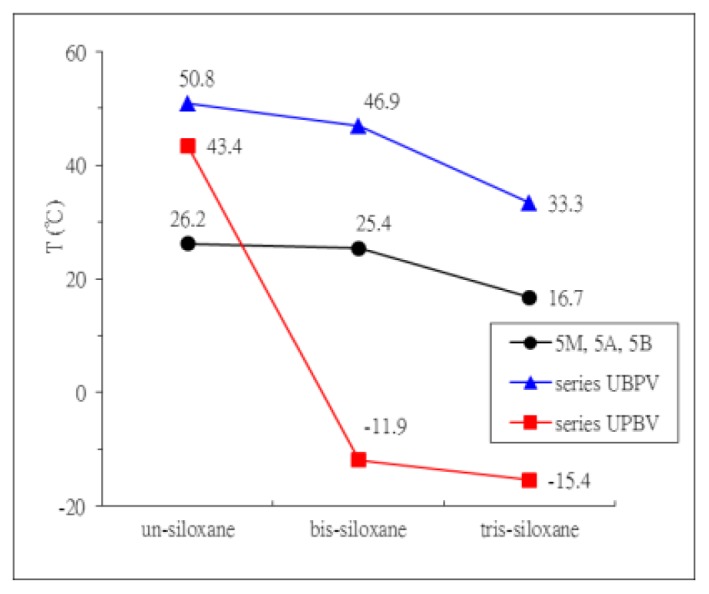
The phase transition temperatures of 5M, 5A, 5B, series UBPV, and series UPBV, which were measured during the cooling process. * chiral center.

**Figure 7:**
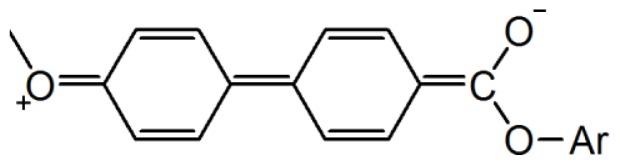
The resonance structure of biphenylcarboxylate group.

In comparison of the thermal transitions between these two series of compounds (series UBPV and series UPBV) and pervious synthesized LCs (5M, 5A, and 5B) [17], some results are summarized as follows: (a) Within the same series, the siloxane group inductions made the liquid crystalline temperature range of a liquid crystal compound become wider. As shown in Figure 5, the liquid crystalline temperature range of compound 5M is 27.0 °C, but the liquid crystalline temperature range of compound 5A rises up to 35.2 °C. The liquid crystalline temperature range of compound UBPV is 46.2 °C, but the liquid crystalline temperature range of compound d-Si-UBPV and t-Si-UBPV increases to 110 °C. The liquid crystalline temperature range of compound UPBV is 82.6 °C, but the liquid crystalline temperature range of compound d-Si-UPBV and t-Si-UPBV rises up to 152 °C. Figure 6 shows the phase transition temperature of the nine compounds, measured during the cooling process. There is a trend that the siloxane group inductions also lowered the transition temperature of the liquid crystalline mesophase, as clearly stated in Figure 5. Although the liquid crystalline mesophase of bis-siloxane compound is similar to that of the tris-siloxane compound, the tris-siloxane compound has a lower phase transition temperature than that of the bis-siloxane compound; (b) The three phenyl molecules (series UBPV and series UPBV) formulate a much wider liquid crystalline phase temperature range than the two phenyls molecules (5M, 5A, and 5B) do. As shown in Figure 5, the liquid crystalline temperature range of 5M, 5A, and 5B is about 27.0~36.8 °C; the liquid crystalline temperature range of series UBPV was measured to be about 46.2~114 °C, and the liquid crystalline temperature range of series UPBV was measured to be about 82.6~154 °C. At the same time, three phenyl molecules possess much wider temperature ranges of S_C_* phase than the two phenyl molecules do. For example, the S_C_* liquid crystalline phase temperature range of 5M, 5A, and 5B is about 15.8~28.3 °C, but the liquid crystalline phase temperature range of series UPBV reaches about 100 °C; (c) In the UBPV series and UPBV series, the differences of phenyl ring arrangements for the mesogenic unit result in the greater differences of the liquid crystal phase formation; the S_B_ phase is the main phase for the UBPV series, while the Sc* phase is the main for the UPBV series. Leadbetter, Forst, and Mazid synthesized 4-*n*-alkoxyphenyl-4′-octyloxybiphenyl-4-carboxylate [21–23] which is equipped with a S_B_ phase. In addition, the UBPV series compounds, in this study, possess the same three phenyls arrangement as that of Leadbetter, Forst, and Mazid. In other words, both studies possess the same S_B_ phase. In addition, the structure of biphenylcarboxylate would produce electronic resonance, which results in the increase of dipoles at the sides of the whole molecule and the gravitation among molecules. The biphenylcarboxylate group here could be seen as a charge transfer (CT) molecule, which shows the displacement of the ð-electron cloud. In other words, the biphenylcarboxylate group has an electronic resonance structure (as shown in Figure 7). It can exhibit a large change in dipole moment. Therefore, the increase of dipole induces the molecular interaction, and the standardized arrangement of the S_B_ phase is formed. However, no similar resonance happens in the UPBV series compounds. Therefore, the factor of the differences of resonance may help explain the physical differences of liquid crystalline between the UBPV series compounds and the UPBV series compounds.

## 3. Experimental Section

^1^H-NMR spectra were recorded on a Varian (CA, USA) VXR-300 or Bruker (MA, USA) 300 MHz spectrometer. Thermal transitions and thermodynamic parameters were determined using a Seiko (Tokyo, Japan) SSC/5200 DSC equipped with a liquid nitrogen cooling accessory. Heating and cooling rates were 10 °C/min. Thermal transition reports were collected during the second heating and cooling scans. A Nikon (Tokyo, Japan) Microphot-FX POM equipped with a Mettler (OH, USA) FP 82 hot stage and a FP 80 central processor was used to observe the thermal transitions and analyze the anisotropic textures. Polymerization reactions were traced using a Nicolet (WI, USA) 520 FT-IR spectrometer.

### 3.1. Synthesis

The general synthetic routes of the intermediates and target molecule are shown in Schemes 1,2. The purity and chemical structures of the intermediates and target compounds can be easily verified by TLC and ^1^H-NMR spectroscopy. The synthetic procedures and chemical analyses of each product are described, sequentially, below.

#### 3.1.1. 4-Hydroxyphenyl(2*S*,3*S*)-2-chloro-3-methylvalerate (**1**) and 4-Hydroxybiphenyl-4′-yl(2*S*,3*S*)-2-chloro-3-methylvalerate (**2**)

Compounds **1**,**2** were prepared by the same method. The synthesis of compound **1** is described below.

To a solution of hydroquinone (2.0 g, 18.2 mmol) and (2*S*,3*S*)-2-chloro-3-methylvaleric acid (2.6 g, 17.2 mmol) in dry dichloromethane (100 mL), dry THF (20 mL), *N*,*N*-dicyclohexylcarbodiimide (DCC, 3.7 g, 17.9 mmol) and 4-(*N*,*N*-dimethyl-amino)-pyridine (DMAP, 0.1 g) were added, to react under nitrogen, in a 250 mL two-neck flask. The reaction mixture was stirred for 24 h at room temperature. The solution was filtered and washed with an excess of dichloromethane. The filtrate was washed with water and dried over anhydrous magnesium sulfate. After removal of the solvent, using evaporation under reduced pressure, the residue was purified by column chromatography on silica gel using hexane/ethyl acetate as eluent to yield 2.42 g (58%) of yellow oil liquid. ^1^H-NMR (300 MHz, CDCl_3_, δ, ppm): 0.78–1.04 (m, 6H, −CH_3_), 1.20–2.10 (m, 3H, −CH–CH_2_–CH_3_), 4.42 (d, 1H, −CHCl–COO–), 6.75 (m, 4H, aromatic protons).

Compound **2** was produced as 2.11 g (42%) of yellow solid. *mp* = 91.3 °C. ^1^H-NMR (300 MHz, CDCl_3_, δ, ppm): 0.78–1.04 (m, 6H, −CH_3_), 1.20–2.10 (m, 3H, −CH–CH_2_–CH_3_), 4.38 (d, 1H, −CHCl–COO–), 6.79–7.51 (m, 8H, aromatic protons).

#### 3.1.2. 4-(10-Undecenyloxy)biphenyl-4′-carboxylic acid (**3**)

4-(4′-Hydroxyphenyl)benzoic acid (4 g, 18.7 mmol) was added to a solution of potassium hydroxide (2.1 g, 37.5 mmol) in ethanol (500 mL, 95%). Potassium iodide (0.2 g) was also added and the solution was heated to reflux for 0.5 h. 10-Undecenyl-1-tosylate (6.1 g, 18.9 mmol) was then slowly added and the solution was heated to reflux overnight. The solution was then extracted with 100 mL of dichloromethane and 50 mL 6N HCl. The extraction solution was dried over anhydrous magnesium sulfate. After removal of the solvent by evaporation under reduced pressure, the residue was recrystallized from ethanol to yield 2.94 g (43%) of white crystal. The remaining white solid *mp* = 165.5 °C. ^1^H-NMR (300 MHz, CDCl_3_, δ, ppm): 1.27–2.10 (m, 16H, − (CH_2_)_8_–), 3.96 (t, 2H, −CH_2_O–), 5.02 (m, 2H, CH_2_=CH–), 5.84 (m, 1H, CH_2_=CH–), 6.90 (m, 2H, aromatic protons), 7.53 (m, 4H, aromatic protons), 8.02 (m, 2H, aromatic protons).

#### 3.1.3. Ethyl-4-(10-undecenyloxy)benzoate (**4**)

Ethyl-4-hydroxybenzoate (2.0 g, 0.012 mol), 10-undecenyl-1-tosylate (3.9 g, 0.012 mol), and potassium carbonate (8.0 g, 0.058 mol) were dissolveted in 100 mL of anhydrous ethylmethylketone in a 250 mL two-neck flask. The solution was heated to reflux for 48 h. The solution was cooled and filtrated. Following the solvent being evaporated, the residue was purified by column chromatography (silica gel, using *n*-hexane/ethyl acetate as an eluent) to yield 3.24 g (85%) of oil liquid. ^1^H-NMR (300 MHz, CDCl_3_, δ, ppm): 1.27–2.10 (m, 19H, − (CH_2_)_8_– and −CH_2_–CH_3_), 3.96 (t, 2H, −CH_2_O–), 4.03 (t, 2H, −COO–CH_2_–), 5.02 (m, 2H, CH_2_=CH–), 5.84 (m, 1H, CH_2_=CH–), 6.86 (m, 2H, aromatic protons), 7.83 (m, 2H, aromatic protons).

#### 3.1.4. 4-(10-Undecenyloxy)benzoic acid (**5**)

Ethyl-4-(10-undecenyloxy)benzoate (1.53 g, 4.8 mmol) was added to a solution of sodium hydroxide (1.2 g, 0.030 mol) in methanol (30 mL, 90%). The solution was cooled and poured into 10 mL of 6 N HCl, and the solution was then extracted with 30 mL of dichloromethane. The extraction solution was dried over anhydrous magnesium sulfate. Following removal of the solvent by evaporation under reduced pressure, the residue was purified by recrystallization from ethanol to yield 1.32 g (95%) of white crystal. ^1^H-NMR (300 MHz, CDCl_3_, δ, ppm): 1.26–1.74 (m, 14H, − (CH_2_)_7_–), 1.96–2.01 (m, 2H, −CH_2_–CH=), 3.96 (t, 2H, −CH_2_O–), 5.02 (m, 2H, CH_2_=CH–), 5.84 (m, 1H, CH_2_=CH–), 6.86 (m, 2H, aromatic protons), 7.96 (m, 2H, aromatic protons).

#### 3.1.5. 4-[4-(10-Undecenyloxy)biphenyl-4′-carbonyloxy]phenyl(2*S*,3*S*)-2-chloro-3-methylvalerate (**UBPV**) and 4-[4-(10-Undecenyloxy)phenyl-4′-carbonyloxy]biphenyl(2*S*,3*S*)-2-chloro-3-methylvalerate (**UPBV**)

Compounds **UBPV**,**UPBV** were prepared by the same method. The synthesis of compound **UPBV** is described below.

4-Hydroxybiphenyl-4′-yl(2S,3S)-2-chloro-3-methylvalerate (**2**) (1.1 g, 3.4 mmol) and 4-(10-undecenyloxy)benzoic acid (**5**) (1.0 g, 3.4 mmol) dissolved in dry dichloromethane (100 mL), *N*,*N*-dicyclohexylcarbodiimide (DCC, 0.85 g, 4.1 mmol) and 4-(*N*,*N*-dimethyl-amino)-pyridine (DMAP, 0.1 g) were added to react under nitrogen. The reaction mixture was stirred for 48 h at room temperature. The solution was filtered and washed with an excess of dichloromethane. The filtrate was washed with water and dried over anhydrous magnesium sulfate. After removal of the solvent by evaporation under reduced pressure, the residue was purified by column chromatography on silica gel using hexane/ethyl acetate as eluent to yield 1.35 g (67%) of white crystal. mp=44.2 °C. ^1^H-NMR (300 MHz, CDCl_3_, δ, ppm): 0.78–2.20 (m, 25H, −CH_3_, − (CH_2_)_8_–, −CH–CH_2_–CH_3_), 4.02 (t, 2H, −CH_2_O–), 4.40 (d, 1H, −CHCl), 5.01 (m, 2H, =CH_2_), 5.80 (m, 1H, CH_2_–CH=), 6.92–8.18 (m, 12H, aromatic protons).

Compound **UBPV** was obtained as white solids (1.17 g, 47%). mp = 55.8 °C. ^1^H-NMR (300 MHz, CDCl_3_, δ, ppm): 0.78–2.20 (m, 25H, −CH_3_, − (CH_2_)_8_–, −CH–CH_2_–CH_3_), 4.02 (t, 2H, −CH_2_O–), 4.42 (d, 1H, −CHCl), 5.01 (m, 2H, =CH_2_), 5.80 (m, 1H, CH_2_–CH=), 6.98–8.21 (m, 12H, aromatic protons).

#### 3.1.6. Liquid Crystal Siloxane Compounds d-Si-UBPV, t-Si-UBPV, d-Si-UPBV, and t-Si-UPBV

Siloxane dimer synthesized from UBPV and UPBV are marked as d-Si-UBPV and d-Si-UPBV; siloxane trimer from UBPV and UPBV are marked as t-Si-UBPV and t-Si-UPBV. Compounds d-Si-UBPV, t-Si-UBPV, d-Si-UPBV, and t-Si-UPBV were prepared using the same method. The synthesis of compound t-Si-UPBV is described below.

To a degassed solution of compound UPBV (0.5 g, 0.8 mmol) and 1,1,1,3,3,5,5-heptamethyltrisiloxane (0.21 g, 0.9 mmol) in 10 mL of dry, freshly distilled toluene was added divinyltetramethyldimethylsiloxyl (2.5 mg) as catalyst. The reaction mixture was stirred at 70 °C under nitrogen for 48 h. Following the solvent being evaporated, the residue was purified by column chromatography (silica gel, using *n*-hexane/ethyl acetate as an eluent) to yield 0.30 g (69%) of white crystal. ^1^H-NMR (300 MHz, CDCl_3_, δ, ppm): 0.0 (m, 21H, −Si–CH_3_), 0.53 (m, 2H, −Si–CH_2_–CH_2_–), 0.78–2.20 (m, 27H, −CH_2_–, −CH_2_–CH_3_ , and −CH–CH_3_), 4.02 (t, 2H, −O–CH_2_–), 4.40 (d, 1H, −CHCl–), 6.92–8.18 (m, 12H, aromatic protons).

## 4. Conclusions

The induction of the soft siloxane molecules is helpful to change the liquid crystalline phase and shift the liquid crystalline temperature. It also broadens the liquid crystal temperature range of S_C_* without inducing chain crystallization phenomenon and diluting the *Ps* value, simultaneously. The siloxane molecule induction is helpful to the smectic phase formation and smectic phase stabilization such as the S_C_* and S_B_ phases. The mesogenic units of three phenyl molecules (series UBPV and series UPBV) formulate much wider liquid crystalline phase temperature range than two phenyl (5M, 5A and 5B) do. The phenyl arrangement differences of mesogenic units result in the greater differences of the liquid crystal phase formation; the S_B_ phase is the head of the UBPV series, while the S_C_* phase is the head of the UPBV series.

## Figures and Tables

**Table 1:** Thermal transitions of compounds UBPV, d-Si-UBPV and t-Si-UBPV.

Compound	Phase transitions, °C (corresponding enthalpy changes, Kcal/mole)

	$\frac{heating}{cooling}$
UBPV	$\frac{K\;55.8\;(3.21)\;S_{\text{A}}\;105{\left(-\right)}^{\text{a}}\;I}{I\;97.0(-)\;S_{\text{A}}\;60.1(-0.14)\;S_{\text{B}}\;50.8(-2.49)K}$
d-Si-UBPV	$\frac{K\;55.0\;(2.27)\;S_{\text{B}}\;153(0.07)\;S_{\text{A}}\;159(1.16)\text{I}}{I\;157(-1.21)\;S_{\text{A}}\;151(-0.07)\;S_{\text{B}}\;46.9(-1.95)K}$
t-Si-UBPV	$\frac{K\;40.2\;(1.84)\;S_{\text{B}}\;143(0.14)\;S_{\text{A}}\;150(0.68)\;\text{I}}{I\;147(-0.50)\;S_{\text{A}}\;141(-0.10)\;S_{\text{B}}\;33.3(-1.51)\;K}$

K = crystal; S_A_ = smectic A; S_B_ = smectic B; I=isotropic.

a determined by optical polarizing microscopic observation.

**Table 2:** Thermal transitions of compounds UPBV, d-Si-UPBV and t-Si-UPBV.

Compound	Phase transitions, °C (corresponding enthalpy changes, Kcal/mole)

	$\frac{heating}{cooling}$
UPBV	$\frac{K\;44.2\;(2.03)\;S_{\text{A}}\;118(0.07)\;N*129(0.13)\;\text{I}}{I\;126(-0.17)N*114(-0.03)\;S_{\text{A}}\;43.4(-0.88)\;K}$
d-Si-UPBV	$\frac{K\;12.6\;(1.05)\;S_{X}44.0(0.37)\;{S_{C}}^{*}144(2.14)\;\text{I}}{I\;140(-1.98)\;{S_{C}}^{*}40.1(-0.26)\;S_{X}-11.9(-0.63)\;K}$
t-Si-UPBV	$\frac{K\;9.4\;(1.29)\;S_{X}42.7(0.08)\;{S_{C}}^{*}142(2.40)\;\text{I}}{I\;139(-1.95)\;{S_{C}}^{*}38.4(-0.12)\;S_{X}-15.4(-0.56)\;K}$

K = crystal; S_C_^*^ = chiral smectic C; *S_X_* = smectic phase; N*=chiral nematic phase; I=isotropic.
